# Impacts of increasing typhoons on the structure and function of a subtropical forest: reflections of a changing climate

**DOI:** 10.1038/s41598-017-05288-y

**Published:** 2017-07-07

**Authors:** Kuo-Chuan Lin, Steven P. Hamburg, Lixin Wang, Chin-Tzer Duh, Chu-Mei Huang, Chung-Te Chang, Teng-Chiu Lin

**Affiliations:** 1 Taiwan Forestry Research Institute, 53 Nan-Hai Road, Taipei, 10066 Taiwan; 2 Environmental Defense Fund, 18 Tremont Street, Boston, Massachusetts United States; 3 Department of Earth Sciences, Indiana University-Purdue University Indianapolis, Indianapolis, IN 46202 USA; 4 Department of Geography, National Taiwan University, No 1 Section 4, Roosevelt Road, Taipei, 10617 Taiwan; 5 Department of Life Science, National Taiwan Normal University, No 88 Section 4, Ting-Chow Road, Taipei, 11677 Taiwan

## Abstract

Due to their destructive and sporadic nature, it is often difficult to evaluate and predict the effects of typhoon on forest ecosystem patterns and processes. We used a 21-yr record of litterfall rates to explore the influence of typhoon frequency and intensity, along with other meteorological variables, on ecosystem dynamics in a subtropical rainforest. Over the past half century there has been an increasing frequency of strong typhoons (category 3; >49.6 m s^−1^; increase of 1.5 typhoons/decade) impacting the Fushan Experimental Forest, Taiwan. At Fushan strong typhoons drive total litterfall mass with an average of 1100 kg ha^−1^ litterfall typhoon^−1^. While mean typhoon season litterfall has been observed to vary by an order of magnitude, mean litterfall rates associated with annual leaf senescence vary by <20%. In response to increasing typhoon frequency, total annual litter mass increased gradually over the 21-year record following three major typhoons in 1994. Monthly maximum wind speed was predictive of monthly litterfall, yet the influence of precipitation and temperature was only evident in non-typhoon affected months. The response of this subtropical forest to strong typhoons suggests that increasing typhoon frequency has already shifted ecosystem structure and function (declining carbon sequestration and forest stature).

## Introduction

Litterfall production and decomposition are important ecosystem processes closely linked to terrestrial ecosystem structure and function e.g., primary productivity^1, 2^. Across ten Amazonian tropical forests, net primary productivity (NPP) was consistently about three times of total litterfall^3^, and climate was a key factor influencing both temporal and spatial patterns of litterfall. A study of litterfall among 34 Finnish Scots pine forests reported that climate factors explained 70% of the variation in inter-site litterfall^4^. Among 64 European forests, evapotranspiration, which is largely determined by climate, was the most important factor explaining litterfall variation within conifer forests^5^. The close relationship between climate and litterfall implies that changes in global climate could have profound effects on litterfall and therefore NPP and carbon sequestration.

Tropical cyclones (hurricanes and typhoons) are extreme events that have a significant impact on litterfall rates and biogeochemical processes^6–10^. Tree mortality and defoliation associated with tropical cyclones are major causes of large litterfall events^6, 11, 12^. Following a tropical cyclone-induced litterfall pulse, litterfall rates typically decline; for how long depends on the magnitude of the damage and the associated recovery rate. Because of the close relationship between litterfall rates and NPP, the recovery of litterfall to pre-cyclone levels has been used as an indicator of ecosystem recovery^13–15^.

Gradual changes in temperature and precipitation, the most widely studied aspects of climate change, can also have major impacts on litterfall through their effects on NPP. In Eurasia where average temperatures are low, increases in average temperature was reported to have a larger positive impact on annual litterfall production than have increases in precipitation^16^. The long-term litterfall data from a Finnish Scots pine forest showed that July temperatures were positively correlated with annual litterfall rates^17^.

In addition to temperature and precipitation, changes in the intensity and frequency of extreme weather events can also have major impacts on key ecosystem processes, including litterfall^18, 19^. Several recent studies indicate that the number of intense tropical cyclones are likely to increase and other studies suggest an overall increase in tropical cyclone frequency^20–23^. Few studies have examined the effect of changes in tropical cyclone frequency and intensity on forests and litterfall in particular. A study in the Northwest Pacific indicates that intense tropical cyclones disproportionally influence litterfall rates^11^, suggesting that any change in cyclone frequency and/or intensity could impact the structure and function of the impacted forests.

For disturbances with return intervals longer than a few decades, such as fire and hurricanes (tropical cyclones originating in the Atlantic), their effects on litterfall dynamics are hard to observe given the paucity of measurements and the period of observed climate changes^24, 25^. Observing long-term ecological effects of extreme events requires long-term data on ecological patterns and processes that span numerous extreme events. The long return intervals between cyclones in most regions of the world make acquisition of such a record challenging if not impossible. Examining cyclone disturbance impacts on ecosystem dynamics in regions with frequent cyclone disturbances of varying intensity provides an opportunity to understand the impacts of changing intensities and frequencies on ecosystem structure^26, 27^. In addition, small or frequent disturbances could have very different ecological effects from large or rare ecosystem disturbances^28^, yet most cyclone studies focus on large, rare and often-catastrophic cyclone events. Thus, long-term data that captures the impacts of cyclones of varying intensities is critical for developing a comprehensive understanding of disturbance-ecosystem interactions.

Many studies report global shifts in patterns of cyclone activities (e.g., from lower to higher latitudes^29–31^) such that regions currently experiencing infrequent cyclone disturbance may face increasing cyclone frequency or intensity in coming decades. By studying the impacts of cyclone disturbances on ecosystem processes in regions currently experiencing frequent cyclone disturbance it should be possible to project ecological consequences for regions experiencing a change in cyclone frequency.

**Figure 1:**
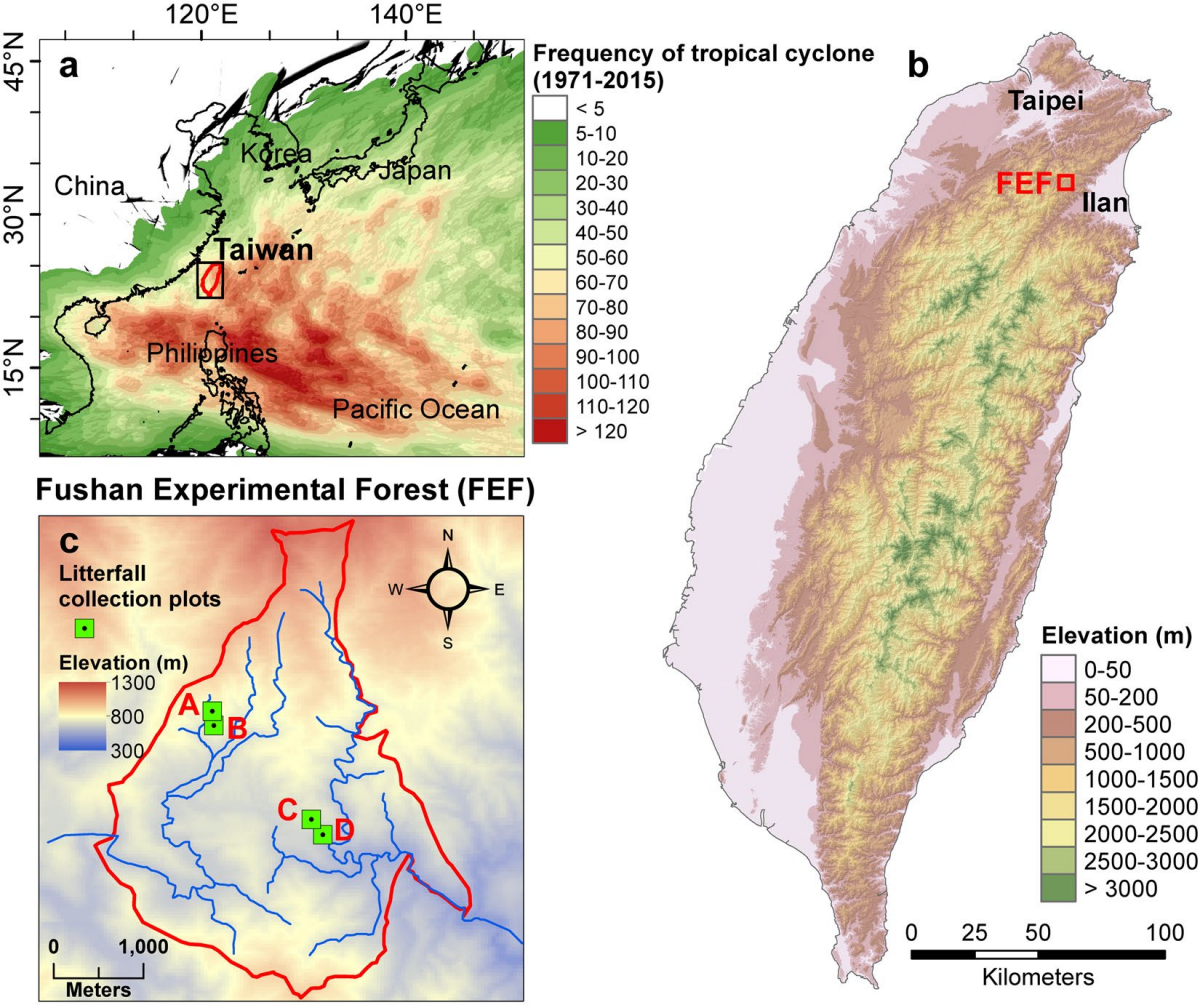
Location map of the Fushan Experimental Forest (FEF). The frequency of tropical cyclones in Northwest Pacific (**a**), location of FEF (**b**), and litterfall collection plots within FEF (**c**). The countries’ boundary map was generated from the map database in ArcGIS v. 10.3^53^. The frequency of tropical cyclone was presented in ArcGIS based on the grid map acquired from Joint Typhoon Warning Center (JTWC)^54^. The elevation maps of FEF and Taiwan (in background) were created based upon 20 m digital elevation model (DEM) obtained from Data.GOV.TW^55^. The litterfall collection plots were built and plotted using Tools in ArcGIS by their coordinates.

Taiwan, a subtropical island of 36 000 km^2^ located in the Northwest Pacific, experiences among the highest frequency of tropical cyclones globally^25^ (Fig. 1a), making it an ideal site for examining the relative influence of climate factors (precipitation and temperature) and extreme events (tropical cyclones) on ecosystem processes. On average around three typhoons make landfall annually in Taiwan, with approximately 60% being ≥ Saffir-Simpson category 3 events^25, 32^. Since 1992 litterfall has been collected on a monthly basis at the Fushan Experimental Forest (FEF) in northeastern Taiwan, and between 1992 and 2012 the forest has been impacted by more than 40 typhoons. We used this dataset to assess temporal patterns of litterfall in relation to typhoon characteristics and other climate variables as well as to consider the potential influence of shifting typhoon frequency and intensity on forest NPP and carbon sequestration. We specifically addressed the following questions.

1. How strongly does typhoon disturbance influence temporal litterfall patterns?
2. Is there evidence that a change in typhoon frequency and intensity is likely to affect litterfall rates, NPP and carbon sequestration?
3. What is the relative importance of typhoons versus other major climate variables, precipitation and temperature, on litterfall rates?

## Results

### Temporal pattern of typhoons and major climate variables

Over the last three decades (1981‒2010) 44 typhoons affected FEF, with a mean of 1.5 typhoons per year. Among these typhoons 17 were ≥ category 3, with a mean frequency of 0.57 yr^−1^. During the period examined there were significant increases in both total number and number of major typhoons (≥category 3) affecting FEF, with an increase of approximately 0.4 y^−1^ for all typhoon categories and 0.15 y^−1^ for major typhoons (Fig. S1a).

Annual mean temperature at FEF showed a small but significant decrease over the last two decades (P = 0.045; Fig. S1b), while the number of rainy days increased significantly, 2 days yr^−1^ (Fig. S1c). The number of days with various rainfall intensities (<10 mm, 10−50 mm, 50−130 mm, 130−200 mm and >200 mm), did not change over the two-decade record except for an increase in the number of days with rainfall <10 mm (P = 0.044; Fig. S1d).

**Figure 2:**
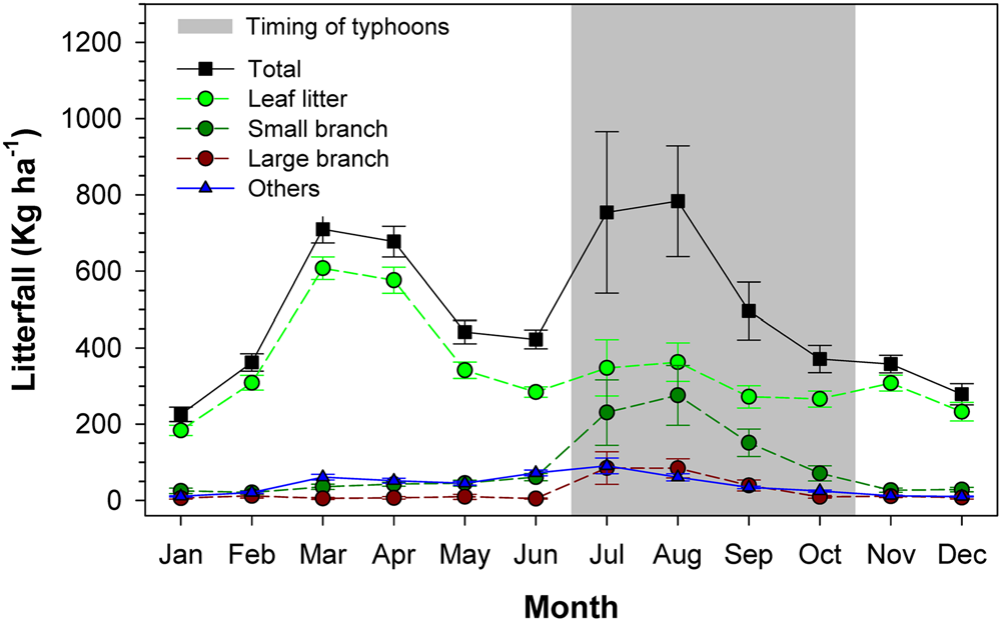
The monthly mean litterfall between 1992 and 2012. The error bars indicate one standard deviation. Gray area indicates typhoon occurring period.

Two annual total litterfall peaks of similar magnitude were observed, one between March and April (spring peak), and one between July and August (and September to a lesser degree, summer peak) (Fig. 2). The spring peak consisted mainly of leaf litter (~80%) with very low inter-annual variation (<20%). In contrast, the summer peak, associated with typhoons, varied by more than an order of magnitude over the 21-year record and the proportional contribution of leaf litter was lower, though still >60% (Fig. 2). Unlike the pattern of total litterfall, there was only one distinct peak in leaf litter mass that occurred between March and April at 1155 ± 280 kg ha^−1^ (Fig. 2).

### Relationship between typhoons and litterfall

**Figure 3:**
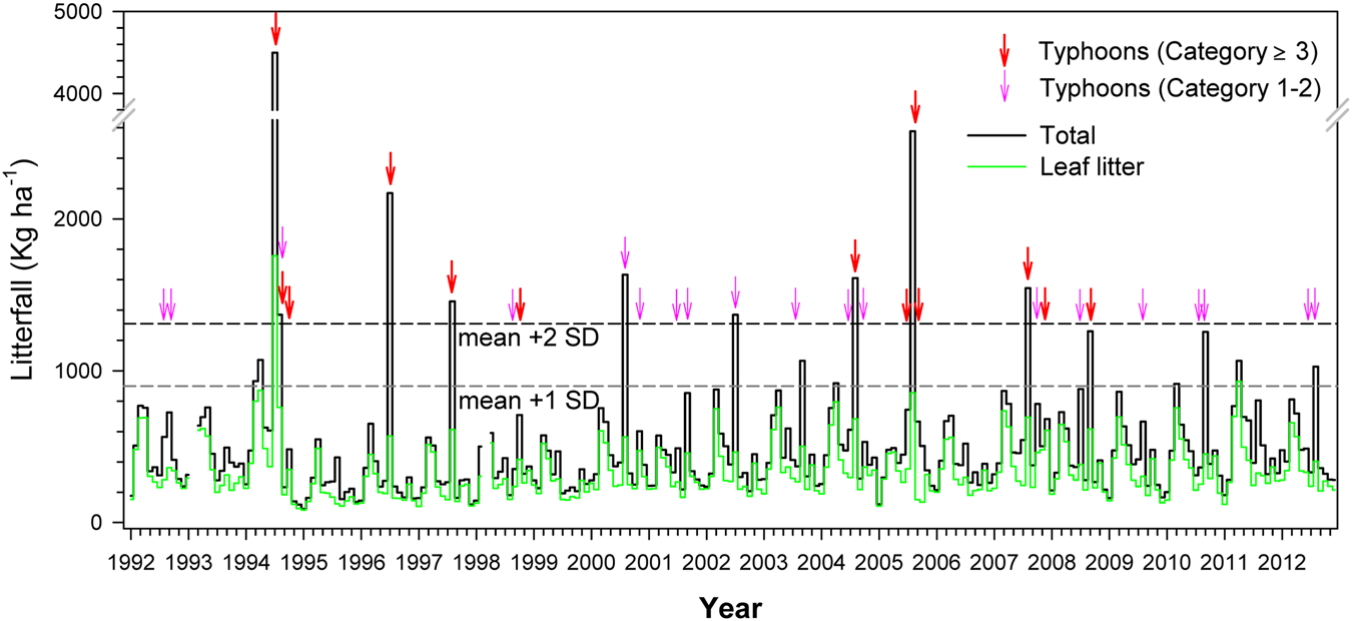
Monthly litterfall during 1992–2012 with highlights of minor and major peaks and typhoons of different intensity categories based on Saffir-Simpson scale. Minor peaks and major peaks refer to monthly total litterfall greater than long-term monthly mean (490 kg ha^−1^) plus one standard deviation (410 kg ha^−1^) and two standard deviations.

We observed three extreme litterfall events (>2 000 kg ha^−1^), all were associated with major typhoons (Fig. 3), as well as six major events (>mean ± 2 SD, or 1 310 kg ha^−1^) that were associated with five major typhoons and two mild typhoons in years that had no major typhoon (Fig. 3). Five major typhoons were not associated with major litterfall events (Fig. 3). Three of them occurred after other major typhoons in the same year and one was associated with a litterfall event that fell slightly short of the cut off for a major event (Fig. 3). The one that was not associated with a litterfall event (Typhoon Zeb in 1998) was associated with a typhoon that did not make landfall and barely met the criteria of a typhoon that affected FEF (typhoons with the center passing within 100 km from FEF, see Methods).

**Figure 4:**
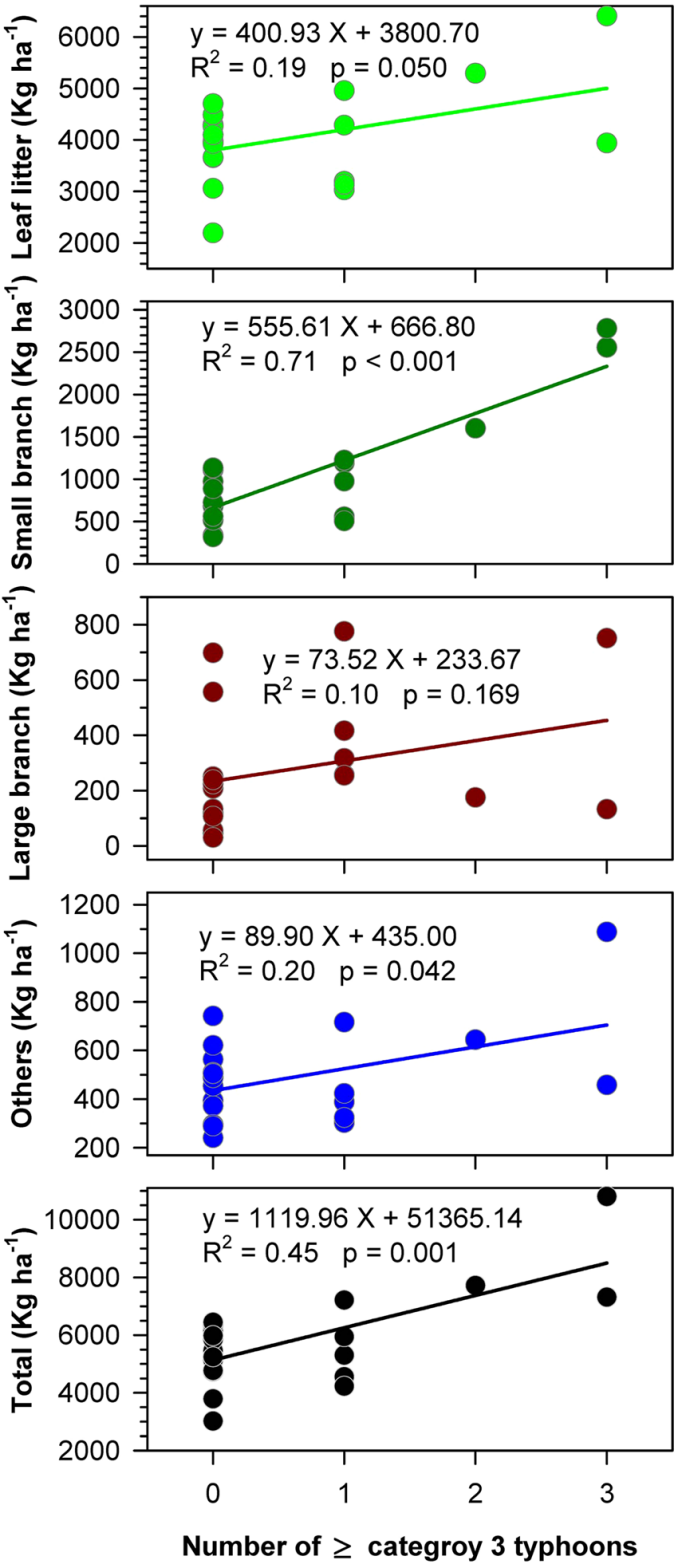
The relationships between annual litterfall components and the number of ≥ category 3 typhoons.

There was a significant positive relationship between number of typhoons and litterfall mass in each year (P <0.05 for total and all components except large branches, Fig. 4). The number of major typhoons explained 45% of the inter-annual variability in total litterfall and 71% of the variability of small branches (Fig. 4), yet adding the number of mild typhoons did not improve the explanatory power of the regression models. The models suggest that the influence of each major typhoon leads to 1 100 kg ha^−1^ of additional litterfall, with approximately 50% of it from small branches. In contrast to total litterfall and small branches, inter-annual variability of leaf litter was better explained by total number of typhoons as compared to major typhoons alone. On average each additional typhoon added 540 kg ha^−1^ of leaf litter (R^2^ = 0.46, P < 0.001).

### Relationship between major climate variables and litterfall

Wind_max_ was a significant predictor in the ARIMA models for total litterfall and all litterfall components of the entire dataset (Table S1), and there was no time lag between Wind_max_ and litterfall based on the cross-correlation analysis (Table S2). Temp_mean_ was also a significant predictor for total litterfall and all litterfall components except large branches (Table S1), and there was a one-to-three month lag in the relationship between temperature and litterfall (Table S2). Rain_max_ was a significant predictor for total litterfall and all litterfall components except flowers and fruits (i.e., the “others” component) (Table S1) and there was no time lag in the relationship (Table S2). The ARIMA models fit the 1992−2005 data well, with R^2^ ranging from 0.77 (leaf litter) to 0.98 (large branches; Table S1 and Fig. S2). The comparisons between model predictions and observed values for the 2006−2012 period were strong, with R^2^ (NSE) ranging from 0.71 (0.70) for others to 0.83 (0.77) for small branches, and the RMSE was only slightly higher than for the fitted results (i.e., 1992−2005) (Fig. S2).

When typhoon-affected months were excluded, Wind_max_ was no longer a significant predictor for any of the litterfall components and only Temp_mean_ and Rain_max_ were significant predictors (Table S3). Temp_mean_ was a significant predictor for all litterfall components and there was a time lag (1−3 moths) between temperature and litterfall (Table S2). Rain_max_ was a significant predictor for leaf litter, small branches and total litterfall (Table S3). The ARIMA models also fit the 1992−2005 data reasonably well when typhoon-affected months were excluded, with R^2^ ranging from 0.74 (total) to 0.97 (large branch; Table S3 and Fig. S3). The R^2^ (NSE) for observed against modeled values during 2006−2012 ranged from 0.64 (0.62) for large branches to 0.86 (0.78) for leaf litter (Fig. S3), supporting the validity of the models.

### Litterfall patterns in relation to the 1994 typhoon season

**Figure 5:**
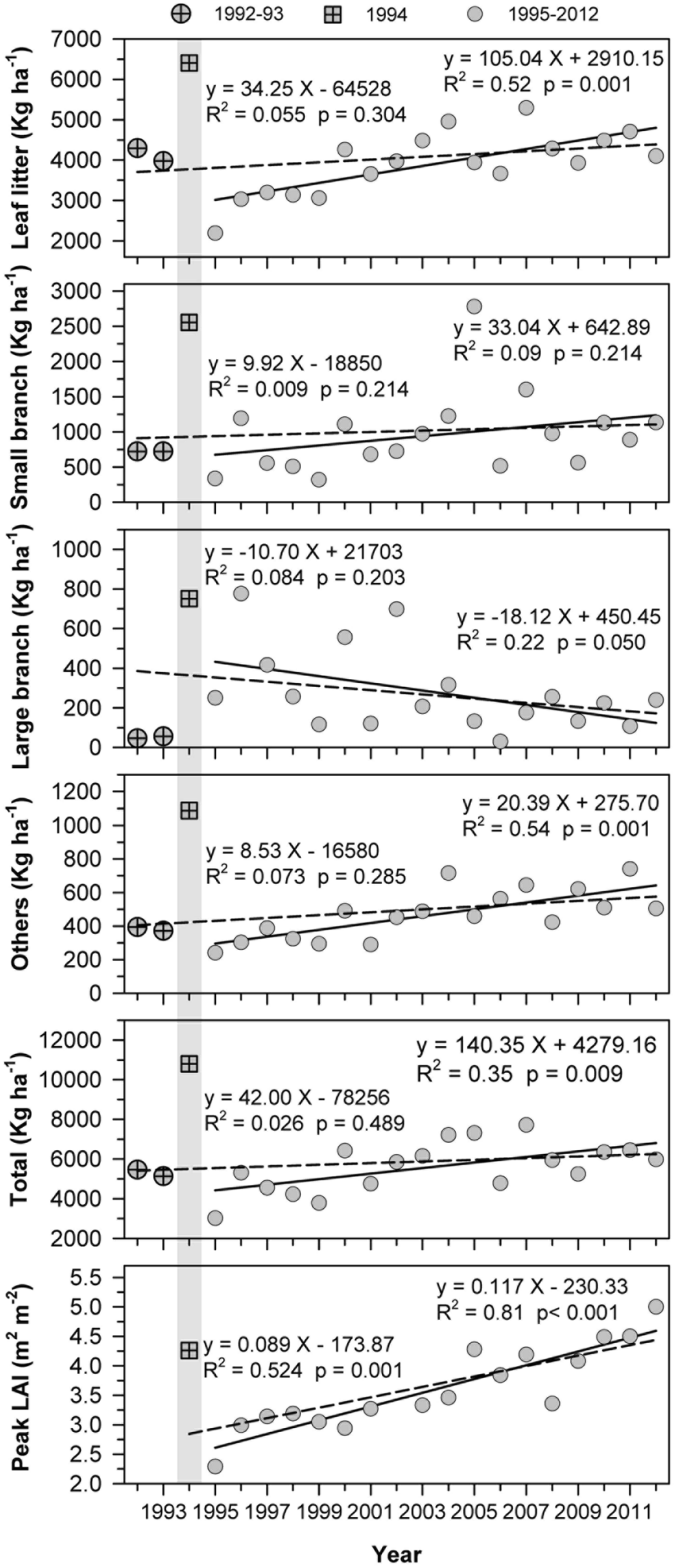
The patterns of annual litterfall and annual peak leaf area index (LAI) at Fushan Experimental Forest. Dash line includes the entire 21-yr data set and the solid includes only data following the 1994 typhoon season (gray area) in which three category 3 typhoon affected the site.

Nineteen ninetyfour is unique in our 21-year record in that it had the highest annual litterfall (10 800 kg ha^−1^) and a record high number of typhoons (3 major and one mild). It took two years for total litterfall mass to increase to pre-1994 levels. Leaf litter mass took six years to increase to pre-1994 levels and annual peak LAI took 10 years to increase to pre-1994 levels (Fig. 5). Over the 1995−2012 period there were significant increases in total litterfall, leaf litter, and flowers and fruits (i.e., the “other” component) and decreases of large branches, with considerable fluctuation year to year (Fig. 5). However, when examining the entire time series of 1992−2015 there were almost no temporal trends evident among components of litterfall as a result of the effects of the 1994 typhoons (Fig. 5), indicating a significant legacy effect of years with frequent and unusually large typhoons. Both peak LAI (Fig. 5) and the number of typhoons (Fig. S1) increased significantly in the years following the 1994 typhoon season, but the result of multiple regression analysis indicates that only peak LAI was a significant predictor of leaf litter mass (R^2^ = 0.53, P = 0.005). The number of typhoons was a significant predictor of small branch mass (R^2^ = 0.55, P = 0.004) and both peak LAI and number of typhoons were significant predictors of total litterfall mass (R^2^ = 0.67, P < 0.001).

## Discussion

### Typhoons dominated litterfall fluctuation

Typhoon disturbance clearly dominated temporal fluctuations of litterfall at FEF over two decades. The importance of typhoon disturbance on litterfall dynamics is also evident in the role that Wind_max_ played in predicting litterfall when typhoon affected months were excluded from the cross-correlations and ARIMA models, shifting from the most important predictor to providing no predictability. We observed increases in both typhoon frequency and litterfall. This positive relationship between the numbers of ≥ category 3 typhoons and litterfall combined with the increase in typhoon frequency over the last two decades suggests that the observed increases in litterfall were at least partly due to increases in typhoon frequency.

The loss of a large quantity of leaves (540 kg ha^−1^ typhoon^−1^) and the production of woody litter (550 kg ha^−1^ per category 3 typhoon) have an important impact on NPP and the carbon stock of the living vegetation. This connection has likely led to this typhoon-impacted forest having very low aboveground biomass, 200−300 Mg ha^−1^, as compared to 400−600 Mg ha^−1^ of many tropical and subtropical forests with similar precipitation and temperature but no exposure to regular typhoons^33^. The dominance of typhoon disturbance on litterfall fluctuation highlights that when combined with changes in the frequency of typhoons, typhoons are likely to have increasingly pervasive effects on ecosystem functioning (e.g., carbon sequestration, carbon stocks). At FEF the amount of typhoon induced litterfall averages 1650 kg ha^−1^ per year, approximately 6% of the aboveground biomass (270 Mg ha^−1^). It is unlikely that foliage regrowth will be able to fully keep pace with increasing typhoon-induced foliage losses if the trend of an increase of 4 typhoons per decade continues. Thus, we would predict that the forest would not be able to sequester enough carbon to compensate for the loss in NPP as a result of additional typhoon-induced leaf litter. Our study suggests that shifts in climate extremes, such as typhoon frequency, are likely to have a measureable impact on ecosystem patterns and processes.

### Typhoon disturbance and forest development

The FEF is highly resilient to typhoon disturbance compared to what has been observed in the neotropics. Following typhoons there is rapid recovery of litterfall and canopy leaf area, mostly within one year, with the exception of years with multiple category 3 typhoons. The resilience of the subtropical FEF to typhoon disturbance is also reflected in streamwater chemistry. Following a 1996 category 3 typhoon nitrate concentrations increased three fold but returned to pre-typhoon levels within a week^25^. By contrast streamwater nitrate at the Luquillo Experimental Forest following the 1989 Hurricane Hugo, which was similar in magnitude to the 1996 typhoon at FEF, returned to pre-disturbance levels after 500 days, two orders of magnitude longer than observed at FEF^34^. The high resilience to typhoon disturbance seen at FEF is also observed at a different experimental forest in central Taiwan in which streamwater chemistry took one to three weeks to return to pre-typhoon levels^27^. High resilience is likely common and essential to forests experiencing annual cyclone disturbance. If recovery takes longer than the interval between large disturbances, available nutrients would be rapidly depleted with a concomitant impact on NPP.

Following the intense, but rare 1994 typhoon season, leaf litter took six years to recover and canopy peak LAI recovered in 10 years, partly because the damage associated with the 1994 typhoons was severe and partly because other typhoons affected the forest during the period. These longer recovery times are consistent with the idea that rare disturbance events take longer to recover from. A comparison of the height of trees that experienced typhoon-induced uprooting or bole-snapping trees versus those undamaged suggests that typhoon disturbance creates relatively constant stress on taller trees that drives the short stature of the FEF forest, despite being an old-growth forest^25^. Although it is impossible to do controlled experiments on typhoon impacts, a comparison between FEF and forests in the same region but further inland and thus less impacted by typhoons provides some insight. A forest 10 km from FEF with similar geology, topography, slope aspect and steepness, and prevailing climate regime experiences considerably lower winds. Predictably the FEF had considerably lower canopy height (15 m for the tallest 10% of trees) and biomass (270 Mg ha^−1^) versus the inland site (20 m and 420 Mg ha^−1^)^35^. It seems reasonable to expect that, in the absence of typhoon disturbance, older trees at FEF would be taller and forest aboveground biomass would be greater. On the other hand, if typhoon frequency and/or intensity increase it could further negatively impact the stature, biomass accumulation and carbon sequestration of the FEF.

### Major climate variables and litterfall pattern

The significant positive relationship between litterfall and temperature and rainfall in the cross-correlations and ARIMA models indicates that temperature and precipitation also affect litterfall patterns, though most likely for different reasons. The positive relationship between mean temperature and litterfall likely reflects increased tree growth with higher temperatures, and the time lag reflects the delayed response in LAI and thus litterfall. In contrast, the positive relationship between Rain_max_ and litterfall likely reflects increased damage to trees associated with intense rainfall, which is consistent with the lack of a time lag in this relationship. There is no evidence that tree growth is water limited; FEF receives an annual average of more than 4000 mm y^−1^ over >200 days^36^.

### Ecological implications

From our long-term litterfall record it appears that the impact and delayed recovery from the 1994 typhoons were unusual. Rare but extreme disturbances such as what we observed in 1994 and have been observed elsewhere, e.g., the 1938 hurricane in northeastern United States, the 1989 Hurricane Hugo in Caribbean, and the 2006 tropical cyclones Monica and Larry in Australia. These events have been the focus of a relatively large number of studies^37–40^. While these major cyclone events do have a large influence on ecosystem patterns and processes, our long-term data indicate that the resulting ecological consequences are not generalizable to more routine cyclone patterns. Cyclone-ecosystem interactions seen through the lens of extreme cyclones must be interpreted with great caution. Even more importantly, impacts on ecosystems that are rarely or only occasionally influenced by cyclones (e.g., eastern US and Puerto Rico, respectively) cannot be extrapolated to ecosystems that are impacted frequently (e.g., Taiwan).

Except for the 1994 extraordinary typhoon season, litterfall and LAI were able to recover in just one year indicating high ecosystem resilience in response “regular” typhoon disturbance^25^. Yet, it would be wrong to infer that forests that are impacted by typhoons annually experience limited effects. Although such forests are capable of recovering from regular typhoon disturbance in one year, as observed at FEF regular typhoon disturbance limits tree growth and stature. Very frequent typhoon disturbances keep the forest from developing the structural characteristics of most old-growth forests (e.g., large trees, low densities); instead, such forests are characterized by short stature and small trees as well as low overall biomass. Frequent typhoon disturbance appears to shift forest development onto a different trajectory, one that could become more common with increased frequency of such disturbances as a result of climate change. If increased cyclone frequency does negatively impact forest carbon stocks there is the potential for positive feedback.

## Methods

### Study site

The study was carried out at the FEF in northeastern Taiwan (24°34″ N, 121°34″ E) (Fig. 1b). The 10 km^2^ FEF is characterized by steep slopes (averaging 35%), and frequent rainfall (>220 days annually)^36^. Its elevation ranges from 670 to 1 400 m. In 1992 an 18 m meteorological tower was installed in a clearing in the experimental forest at 670 m above sea level^36^. Between 1993 and 2007, annual precipitation ranged from 2 900 to 6 650 mm with a mean of 4 240 mm^25^. The annual mean temperature was 18.2 °C with a low monthly average of 11.8 °C in February and a maximum in July of 24.1 °C^36^. Relative humidity at FEF is above 90% throughout most of the year. The forest is characterized as a moist subtropical mixed evergreen forest without an observable dormant season.

Within FEF there are 515 vascular plant species belonging to 329 genera and 124 families^41^. The dominant tree species are: *Castanopsis carlesii* var. *sessilis* Nakai, *Machilus thunbergii* (Sieb. et Zucc.) Kostermans, *Engelhardtia raxburghiana* Wall., *Meliosma squamulata* Hance, *Litsea acuminata* (Blume) Kurata*, Diospyros morrisiana* Hance, *Helicia formosana* Hems, and *Pyrenaria shinkoensis* (Hayata) Keng. Common shrubs are mostly *Ardisia quinquegona* Blume, *Blastus cochinchinensis* Lour, *Lasianthus fordii* Hance, and *Meliosma squamulata* ^42^. The forest is multistoried with scattered tree ferns and shrubs, and with an herbaceous ground cover of 20% on ridges, 70% on slopes and 80% in the valleys. The bedrock at FEF consists of black argillite of the Oligocene. The soils are coarse-loamy Typic Dystochrepts, with the top 30 cm being very acidic (pH 3.8–5.0), and having a low bulk density (0.5–0.8 Mg m^−3^) and low base saturation (2–5%)^43, 44^.

### Typhoon information

From the typhoon database of the Central Weather Bureau, we calculated the number and intensity of typhoons that affected FEF between 1981 and 2010. A typhoon was considered to have affected FEF if the distance between the typhoon center and FEF was <100 km at any time^29^. This criterion is used because the radius of maximum wind of most typhoons is >100 km.

### Litterfall collection

Litterfall was collected in two 20 × 20 m plots (A and B) established in 1991 and two more (C and D) established in 1995 (Fig. 1c). The elevations of these plots ranged from 690 to 780 m, with slopes from 21° to 28°, facing south and southwest. The dominant tree species in the plots were similar, with six species (*Castanopsis cuspidate* var. *carlesii*, *Meliosma squamulata* Hance, *Diospyros morrisiana* Hance, *Machilus thunbergii* Sieb. & Zucc., *Engelhardia roxburghiana* Wall., *Pyrenaria shinkoensis* (Hayata) Keng) accounting for 51−60% of the trees in all four plots. The average diameter at breast height (DBH) of the trees >2 cm was 20 cm (ranging from 16 to 26 cm), mean basal area was 36 m^2^ ha^−1^ (32−51 m^2^ ha^−1^), mean tree height was 11 m (9.4 to 11.6 m), and the mean tree density was 1100 (930−1 500) trees ha^−1^ in 1991 (Table S4). By 2012, mean DBH increased 7% to 22 cm (19−26 cm; P < 0.001) but there was no significant change in tree density or height (Table S4).

Ten 15 cm high, 50 × 50 cm horizontal litterfall traps were established in each plot 1 m above the ground. The sides of the traps were made of wood beveled on the upper edge while the bottoms were constructed of 1 mm mesh nylon netting. In the center of each plot an additional 2 × 5 m 1 mm mesh net was placed on the ground to collect branches with diameters >2 cm. Litterfall was collected once a month and was brought back to the laboratory, dried at 40 °C for 24 h and separated into: foliage, large branches (diameter > 2 cm), small branches (all woody material other than large branches), and other material (mainly flowers and fruits). After litterfall was sorted it was dried to a constant weight at 65 °C. Litterfall was collected from January 1992 and January 1996 through December 2012 for the plots initiated in 1991 and 1995, respectively.

### Leaf area index (LAI) measurements

LAI was measured at 5 m intervals along a 300-m line transect running along a ridge (800 m) starting in April 1994 using a Li-Cor LAI-2000 Plant Canopy Analyzer^25^. Measurements were taken multiple times annually with every effort to obtain LAI readings before and after typhoon disturbances.

### Data Analysis

We used regression models to examine patterns of annual precipitation and temperature. Based on mean monthly litterfall between 1992 and 2012, we defined total monthly litterfall (i.e., total of all litterfall components) greater than mean ± 1 SD (standard deviation) but smaller than mean ± 2 SD as minor litterfall peaks and those greater than mean ± 2 SD but less than 2 000 kg ha^−1^ as major peaks. The few months with litterfall greater than 2 000 kg ha^−1^ were considered extreme peaks. We used regression models to examine the relationship between typhoons and litterfall production using the number of typhoons as the independent variable and litterfall as dependent variable. We defined typhoons with an intensity ≥category 3 based on Saffir-Simpson scale (maximum wind speed > 49.6 m s^−1^)^45^ as major typhoons and those of category 1 or 2 were defined as mild typhoons.

The FEF experienced three major typhoons and one mild typhoon in 1994, the maximum number recorded in a single year since record keeping began in 1958. Canopy leaf area index dropped as much as 2/3 on the ridge^25^ and the annual litterfall reached 10 000 kg ha^−1^ compared to only 3 000 kg ha^−1^ in 1993 when no typhoons affected FEF^11^. Using linear regression models we explored recovery of litterfall mass to pre-disturbance levels to infer forest recovery following the unprecedented 1994 typhoon season. We also used a regression model to examine the relationship between the post-1994 annual litterfall and the peak annual LAI. We did not have peak LAI for 2002 because constant rainfall prevented us from taking measurements prior to the typhoon season.

### Cross-correlation and ARIMA models

The relationships between monthly litterfall and climate parameters, 1) monthly minimum temperature (Temp_min_), 2) monthly mean temperature (Temp_mean_), 3) monthly maximum temperature (Temp_max_), 4) monthly rainfall, 5) maximum daily rainfall of each month (Rain_max_), and 6) monthly maximum wind speed (Wind_max_) were examined using cross-correlations with time lags up to 3 months. In addition to using the entire dataset, we also conducted the analysis excluding typhoon-impacted months (40 months). The missing gaps (values for typhoon months) were filled with the long-term mean of the same non-typhoon month and considered as the baseline for this study. This is needed for autoregressive integrated moving average (ARIMA) models^46, 47^.

We built ARIMA models to evaluate the contribution of climate variables (independent variables) on the time series of litterfall (dependent variable)^17, 48^. Because litterfall is highly seasonal, we built seasonal ARIMA models that include both non-seasonal and seasonal autoregressive (p and P), moving average (q and Q) and differencing (d and D) (small letters for non-seasonal and capital letters for seasonal). The pdq and PDQ were iterated to identify the best-fit models and the goodness-of-fit of the models were evaluated through an autocorrelation function (ACF) and partial autocorrelation functions (PACF) of the residuals by the Ljung-Box test with the *p*-value > 0.05. The model with the smallest normalized Bayesian information criterion (BIC) was selected as the best-fit model^49^. The significance of the parameters was determined based on the *t* statistic exceeding 2 in absolute value^50^. The ARIMA models were constructed using data collected during the 1992−2005 period and then validated for 2006−2012 with the predictions compared to the observed values. The differences between predicted and observed values were evaluated by root mean squared error (RMSE), Nash-Sutcliffe efficiency (NSE), and coefficient of determination (R^2^). RMSE assesses the residual between observed and predicted values, and NSE evaluates model efficiency and predictive power, with the value ranging from negative infinity to one, the closer to one the better the model’s performance^51, 52^.

## Electronic supplementary material

Supplementary Information
