# Supplementary material for: Impacts of increasing typhoons on the structure and function of a subtropical forest: reflections of a changing climate
Source: Sci Rep. 2017 Jul 7;7:4911. doi: 10.1038/s41598-017-05288-y (PMC5501816; doi:10.1038/s41598-017-05288-y)
Supplement: Supplementary file 1 — Supplementary Information [file 41598_2017_5288_MOESM1_ESM.pdf]

SUPPLEMENTARY INFORMATION

Impacts of increasing typhoons on the structure and function of a subtropical forest: reflections of a changing climate

Kuo-Chuan Lin[1], Steven P. Hamburg[2], Lixin Wang[3], Chin-Tzer Duh[1], Chu-Mei Huang[1], Chung-Te Chang[4], and Teng-Chiu Lin[5]

[1]Taiwan Forestry Research Institute, 53 Nan-Hai Road, Taipei 10066, Taiwan.

[2]Environmental Defense Fund, 18 Tremont Street, Boston, Massachusetts, United States.

[3]Department of Earth Sciences, Indiana University-Purdue University Indianapolis, Indianapolis, IN 46202, USA.

[4]Department of Geography, National Taiwan University, No 1 Section.4, Roosevelt Road, Taipei 10617, Taiwan.

[5]Department of Life Science, National Taiwan Normal University, No 88 Section 4, Ting-Chow Road, Taipei 11677, Taiwan.

*Corresponding author

Dr. Chung-Te Chang

E-mail: changchuante@gmail.com

Dr. Teng-Chiu Lin

E-mail: tclin@ntnu.edu.tw

Tel: +886-2-77346240

Fax: +886-2-29312904

Supplementary Table S1. Coefficient (β), standard error (SE), and *t* statistics of the parameters of the best ARIMA models (all *p*-value of Ljung-Box test > 0.05) for monthly litterfall between 1992 and 2005.

ARIMA(p, d, q)(P, D, Q)	parameters	β	SE	*t*	*p*-value
Leaf litter					
(0, 0, 3)(1, 0, 1)	AR (1)	0.991	0.010	99.987	< 0.001
$R^2 = 0.77$	MA (1)	0.822	0.087	9.454	< 0.001
	$Temp_{mean}$	11.244	5.812	1.935	0.030
	$Rain_{max}$	0.359	0.083	4.335	< 0.001
	$Wind_{max}$	7.771	1.761	4.412	< 0.001
Small branch					
(0, 0, 0)(2, 0, 2)	Constant	-82.683	13.548	-6.103	< 0.001
$R^2 = 0.97$	AR(1)	-0.687	0.210	-3.280	0.001
	$Temp_{mean}$	3.058	0.687	4.509	< 0.001
	$Rain_{max}$	0.104	0.042	2.501	0.013
	$Wind_{max}$	5.354	0.793	6.750	< 0.001
Large branch					
(0, 0, 0)(0, 0, 1)	$Rain_{max}$	0.010	0.014	2.154	0.033
$R^2 = 0.98$	$Wind_{max}$	0.603	0.134	4.506	< 0.001
Others					
(1, 0, 0)(1, 0, 0)	AR (1)	0.594	0.064	9.249	< 0.001
$R^2 = 0.82$	$Temp_{mean}$	2.440	0.432	5.648	< 0.001
	$Wind_{max}$	1.504	0.253	5.933	< 0.001
Total					
(1, 0, 0)(1, 0, 1)	AR (1)	0.387	0.076	5.106	< 0.001
$R^2 = 0.94$	MA (1)	0.752	0.090	8.322	< 0.001
	$Temp_{mean}$	20.572	3.467	5.934	< 0.001
	$Rain_{max}$	0.463	0.110	4.205	< 0.001
	$Wind_{max}$	9.169	2.393	3.832	< 0.001

Supplementary Table S2. The optimum cross-correlation coefficients ($P < 0.05$) between original monthly climate variables and monthly litterfall and typhoon months replaced by long-term means of the respective months.

	Monthly climate variables v.s. monthly litterfall	Monthly climate variables v.s. litterfall of typhoon months replaced by long-term means
Leaf litter		
$Temp_{mean}$	$0.380_{(3)}$	$0.533_{(3)}$
$Temp_{max}$	$0.298_{(3)}$	$0.435_{(3)}$
$Temp_{min}$	$0.380_{(3)}$	$0.521_{(3)}$
Rainfall	--	--
$Rain_{max}$	$0.182_{(0)}$	$0.144_{(0)}$
$Wind_{max}$	$0.342_{(0)}$	--
Small branch		
$Temp_{mean}$	$0.354_{(0)}$	$0.371_{(0)}$
$Temp_{max}$	$0.317_{(0)}$	$0.306_{(0)}$
$Temp_{min}$	$0.378_{(0)}$	$0.357_{(0)}$
Rainfall	$0.381_{(0)}$	$0.188_{(3)}$
$Rain_{max}$	$0.527_{(0)}$	$0.183_{(0)}$
$Wind_{max}$	$0.711_{(0)}$	$0.451_{(0)}$
Large branch		
$Temp_{mean}$	$0.258_{(0)}$	$0.150_{(0)}$
$Temp_{max}$	$0.222_{(0)}$	--
$Temp_{min}$	$0.295_{(0)}$	$0.191_{(0)}$
Rainfall	$0.319_{(0)}$	$0.158_{(1)}$
$Rain_{max}$	$0.461_{(0)}$	--
$Wind_{max}$	$0.589_{(0)}$	$0.162_{(0)}$
Others		
$Temp_{mean}$	$0.510_{(1)}$	$0.634_{(1)}$
$Temp_{max}$	$0.434_{(1)}$	$0.544_{(1)}$
$Temp_{min}$	$0.511_{(1)}$	$0.631_{(2)}$
Rainfall	$0.264_{(3)}$	$0.276_{(3)}$
$Rain_{max}$	$0.324_{(3)}$	$0.255_{(3)}$
$Wind_{max}$	$0.401_{(0)}$	$0.235_{(1)}$
Total		
$Temp_{mean}$	$0.295_{(1)}$	$0.515_{(3)}$
$Temp_{max}$	$0.289_{(0)}$	$0.411_{(3)}$
$Temp_{min}$	$0.281_{(1)}$	$0.512_{(3)}$
Rainfall	$0.278_{(0)}$	--
$Rain_{max}$	$0.425_{(0)}$	$0.187_{(0)}$
$Wind_{max}$	$0.620_{(0)}$	--

The numbers in the suffix parentheses indicated the time lag (months) and "--" showed no significant correlation between climate variables and litterfall.

Supplementary Table S3. Coefficient (β), standard error (SE), and *t* statistics of the parameters of the best ARIMA models (all *p*-value of Ljung-Box test > 0.05) of monthly litterfall with data of typhoon months replaced by long-term means of the respective months between 1992 and 2005.

ARIMA(p, d, q)(P, D, Q)	parameters	β	SE	*t*	*p*-value
Leaf litter					
(1, 0, 1)(1, 0, 1)	AR (1)	0.707	0.104	6.807	< 0.001
R^2 = 0.75	MA (1)	0.819	0.077	10.586	< 0.001
	$Temp_{mean}$	15.494	4.687	3.306	0.001
	$Rain_{max}$	0.290	0.080	3.606	< 0.001
Small branch					
(1, 0, 1)(1, 0, 1)	Constant	-52.089	9.157	-5.686	< 0.001
R^2 = 0.83	AR (1)	0.632	0.172	3.679	< 0.001
	$Temp_{mean}$	2.499	0.411	6.076	< 0.001
	$Rain_{max}$	0.009	0.024	2.767	0.038
Large branch					
(0, 0, 0)(2, 1, 0)	AR (1)	-0.838	0.078	-10.746	< 0.001
R^2 = 0.97	$Temp_{mean}$	0.061	0.084	2.788	0.038
Others					
(1, 0, 0)(1, 0, 1)	AR (1)	0.499	0.071	7.039	< 0.001
R^2 = 0.81	MA (1)	0.915	0.120	7.619	< 0.001
	$Temp_{mean}$	2.305	0.665	3.466	0.001
Total					
(1, 0, 0)(1, 0, 1)	AR (1)	0.473	0.072	6.597	< 0.001
R^2 = 0.74	MA (1)	0.810	0.085	9.500	< 0.001
	$Temp_{mean}$	19.111	5.588	3.420	0.001
	$Rain_{max}$	0.359	0.105	3.427	0.001

Supplementary Table S4. Forest characteristics (mean ± standard error) of the four plots in which litterfall was collected within the Fushan Experimental Forest. (Plot A and B were sampled since January 1991 and Plot C and D were sampled since January 1995).

Plot	A	B	C	D
DBH (cm)				
1991–1995	20.4 ± 1.0	19.0 ± 1.1	16.3 ± 1.0	25.7 ± 2.4
2012	22.1 ± 1.05	20.5 ± 1.25	18.6 ± 1.29	26.0 ± 2.15
Tree height (m)				
1991–1995	11 ± 0.3	11 ± 0.4	9 ± 0.5	11 ± 0.5
2012	11 ± 0.1	11 ± 0.1	11 ± 0.2	12 ± 0.2
Tree density (trees ha^{-1})				
1991–1995	980	1500	925	975
2012	910	1350	850	1025

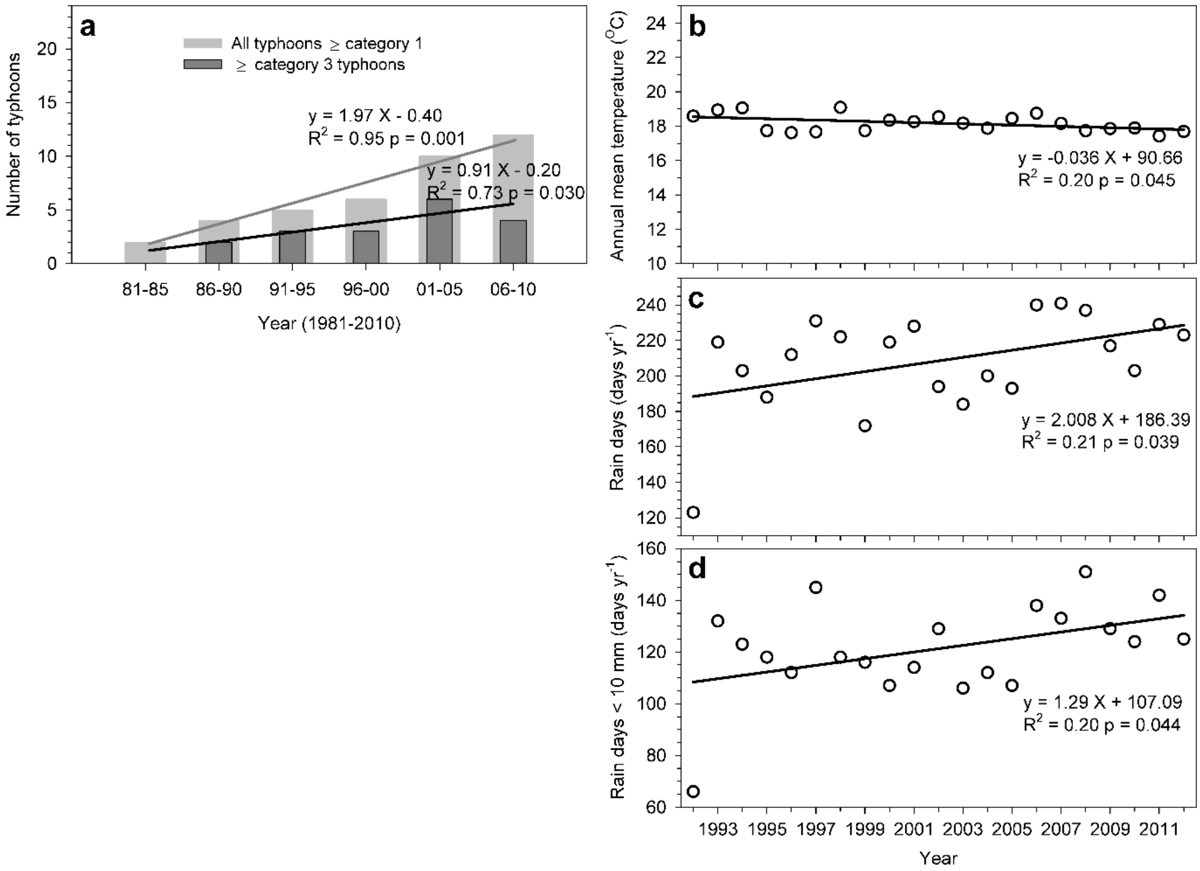

Supplementary Figure S1. The number of land-passing typhoons within 100 km from (a) the Fushan Experimental Forest at 5-year intervals (1981-2010) and the temporal patterns of annual mean temperature (b), annual rain days (c), and annual rain days with rainfall < 10 mm (d)

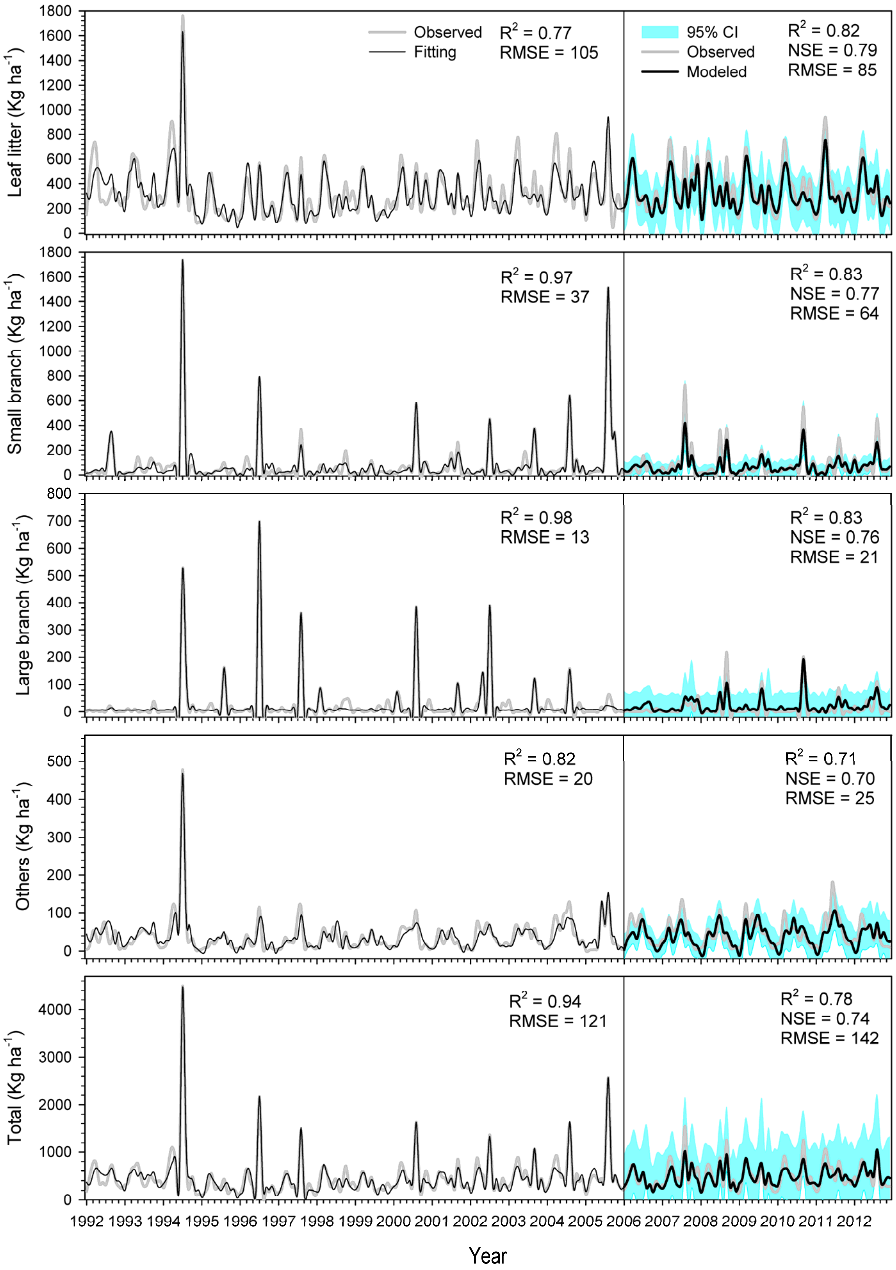

Supplementary Figure S2. The observed and fitting monthly litterfall components by auto-regressive integrated moving average (ARIMA) models (see Table 1) between 1992 and 2005 and observed and modeled monthly litterfall components during 2006-2012 at Fushan Experimental Forest.

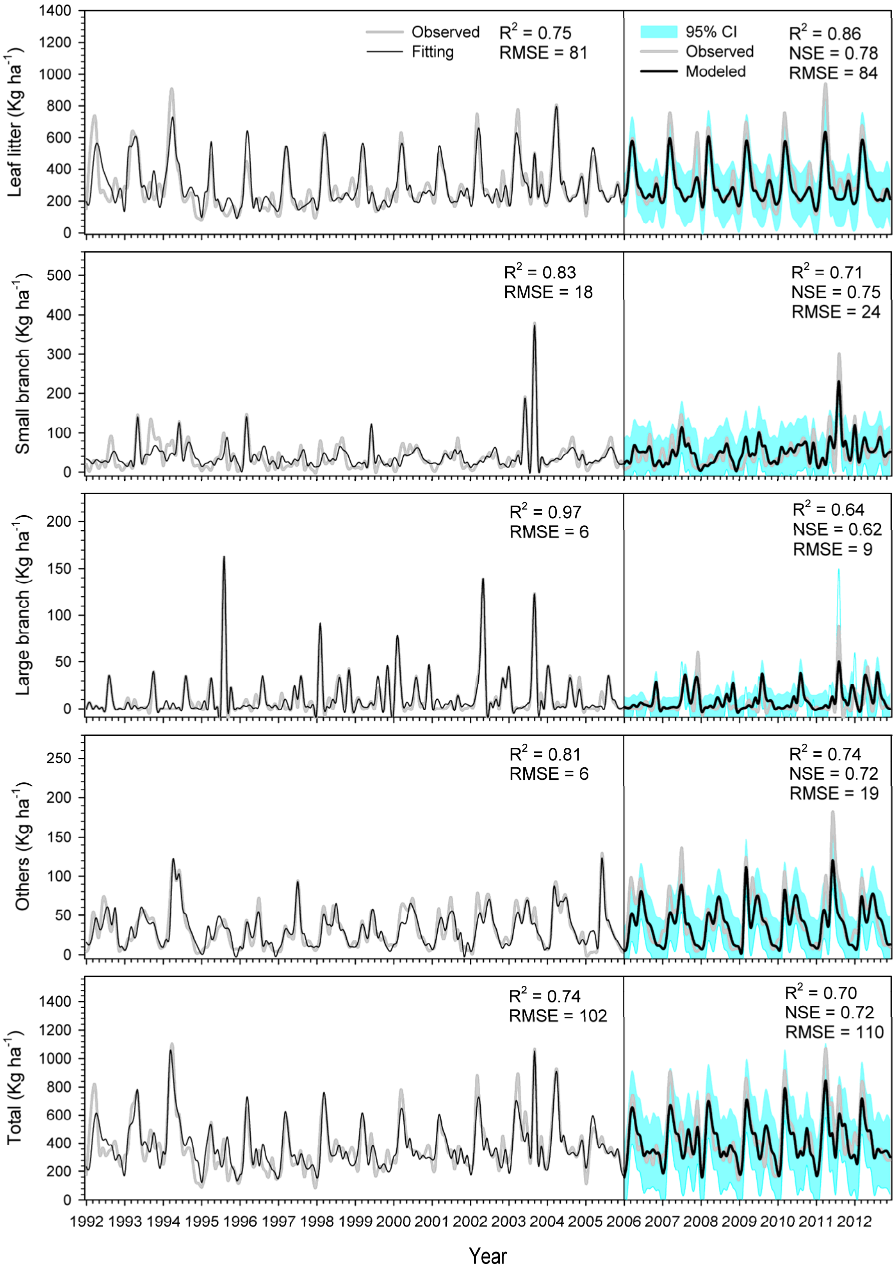

Supplementary Figure S3. The observed and fitting monthly litterfall components by auto-regressive integrated moving average (ARIMA) models (see Table S3) between 1992 and 2005 and observed and modeled monthly litterfall components during 2006–2012 with typhoon excluded data at Fushan Experimental Forest. The typhoon affected month was replaced by the mean of the corresponding months that were not affected by typhoon over the 21 years.
